# Analysis of Two Lysozyme Genes and Antimicrobial Functions of Their Recombinant Proteins in Asian Seabass 

**DOI:** 10.1371/journal.pone.0079743

**Published:** 2013-11-07

**Authors:** Gui Hong Fu, Zhi Yi Bai, Jun Hong Xia, Feng Liu, Peng Liu, Gen Hua Yue

**Affiliations:** 1 Molecular Population Genetics Group, Temasek Life Sciences Laboratory, National University of Singapore, Singapore, Singapore; 2 Department of Biological Sciences, National University of Singapore, Singapore, Singapore; 3 Key Laboratory of Freshwater Aquatic Genetic Resources, Shanghai Ocean University, Ministry of Agriculture, Shanghai, China; University of Sydney, Australia

## Abstract

Lysozymes are important proteins of the innate immune system for the defense against bacterial infection. We cloned and analyzed chicken-type (c-type) and goose-type (g-type) lysozymes from Asian seabass (*Lates calcarifer*). The deduced amino acid sequence of the c-type lysozyme contained 144 residues and possessed typical structure residues, conserved catalytic residues (Glu^50^ and Asp^67^) and a “GSTDYGIFQINS” motif. The deduced g-type lysozyme contained 187 residues and possessed a goose egg white lysozyme (GEWL) domain containing three conserved catalytic residues (Glu^71^, Asp^84^, Asp^95^) essential for catalytic activity. Real time quantitative PCR (qRT-PCR) revealed that the two lysozyme genes were constitutively expressed in all the examined tissues. The c-type lysozyme was most abundant in liver, while the g-type lysozyme was predominantly expressed in intestine and weakly expressed in muscle. The c-type and g-type transcripts were up-regulated in the kidney, spleen and liver in response to a challenge with *Vibrio harveyi*. The up-regulation of the c-type lysozyme was much stronger than that of the g-type lysozyme in kidney and spleen. The recombinant proteins of the c-type and g-type lysozymes showed lytic activities against the bacterial pathogens *Vibrio harveyi* and *Photobacterium damselae* in a dosage-dependent manner. We identified single nucleotide polymorphisms (SNPs) in the two lysozyme genes. There were significant associations of these polymorphisms with resistance to the big belly disease. These results suggest that the c- and g-type genes play an important role in resistance to bacterial pathogens in fish. The SNP markers in the two genes associated with the resistance to bacterial pathogens may facilitate the selection of Asian seabass resistant to bacterial diseases.

## Introduction

Lysozymes are the important antibacterial effector of the innate immunity in animals. They cleave the β-(1,4)-glycosidic bond between N-acetylmuramic acid (NAM) and N-acetylglucosamine (NAG) in the peptidoglycan layer of bacterial cell walls, and [1]. Lysozymes are categorized into six types: chicken-type (c-type) [2], goose-type (g-type) [3], invertebrate-type (i-type) [4], plant-type [5], bacterial type [6] and phage-type [4]. Lysozymes widely exist in numerous organisms ranging from bacteriophages up to plants and animals [7]. In animals, the i-type lysozyme has only been characterized in invertebrates, while the g- and c-type lysozymes are found in both vertebrates and invertebrates [8,9]. The g-type and c-type lysozymes have been identified in some fish species, such as Senegalese sole (*Solea senegalensis*) [10], orange-spotted grouper (*Epinephelus coioides*) [11], large yellow croaker (*Larimichthys crocea*) [12], grass carp (*Ctenopharyngodon idellus*) [13], Japanese flounder (*Paralichthys olivaceus*) [14], turbot (*Scophthalmus maximus*) [15], rainbow trout (*Oncorhynchus mykiss*) [16] and common carp (*Cyprinus carpio* L.) [17]. The expressions of the lysozyme genes were positively regulated by bacterial infection, which suggests an involvement of lysozymes in the anti-microbial defense in fish [12,15]. The recombinant proteins of two types of lysozymes purified from *Escherichia coli* show antimicrobial activity against *Vibrio anguillarum* [14] in Japanese flounder, *Aeromonas hydrophila*, *Vibrio fluvialis*, *Aeromonas sobria* and *Micrococcus lysodeikticus*, in gastropod *Oncomelania hupensis* [18], *V. anguillarum* or *M lysodeikticus* in Mediterranean mussel [19], *V. splendidus* and *V.parahaemolyticus* in Chlamys farreri [8], as well as *Vibrio alginolyticus* and *Vibrio parahemolyticus* in shrimp [20]. 

The Asian seabass (*Lates calcarifer* Bloch, 1790) belonging to the family Latidae, is known also as Barramundi in Australia. It is widely distributed in tropical Indo-west Pacific, and Northern Australia. Since 1998, we started a breeding program for Asian seabass to improve growth [21,22], and developed a number of genomic tools to facilitate the selective breeding program, such as microsatellites [21,23], SNPs in genes [24], BAC and cDNA libraries [25,26], linkage and physical maps [27,28,29], a genetic tracing system [30], and transcriptomes [31]. Diseases are the major bottleneck for sustainable and profitable aquaculture [32]. In Asian seabass, there are several major diseases caused by viruses, bacterial pathogens and parasites. Big belly, also called skinny pot-belly, is a disease caused by a facultative intracellular Gram-negative bacterium [33]. It is present in several South East Asian countries, including Indonesia, Singapore and Malaysia. This disease causes severe clumping of internal organs, abdominal distension and muscular atrophy in Asian seabass fry (< 5 g). The mortality rate of this disease exceeds 80% [33]. Recently, our research has also begun to focus on breeding Asian seabass resistant to diseases. However, little information is available about genes related to disease resistance.

In the present study, in order to investigate the functions of the lysozyme genes against bacterial pathogens we isolated the c-type and g-type lysozyme cDNA and analysed their expression profiles in normal individuals as well as in individuals challenged with *Vibrio harveyi* (*V. harveyi*). With the two types of recombinant lysozymes, we characterized their lytic activity against *V. harveyi*, *Photobacterium damselae* (*P. damselae*) strains and *E. coli* DH5α. In addition, we identified SNPs in the two genes and analysed their associations with resistance against the big belly disease. This study could shed new insights on the defence functions of the c-type and g-type lysozymes again bacterial pathogens and supply DNA markers for selection of Asian seabass resistant to the big belly disease.

## Materials and Methods

### Ethics Statement

All handling of fishes was conducted in accordance with the guidelines on the care and use of animals for scientific purposes set up by the Institutional Animal Care and Use Committee (IACUC) of the Temasek Life Sciences Laboratory, Singapore. The IACUC has specially approved this study within the project “Breeding of Asian seabass” (approval number is TLL (F)-12-004).

### Fishes

Asian seabass individuals were cultured in the Marine Aquaculture Center (MAC), Singapore with the standard operation protocol (SOP) set by MAC. Thirty individuals of healthy Asian seabass at the age of 90 days post-hatch (dph) with an average body weight of 30.05 ± 2.68 g were transported to large tank containing 500 L seawater located in the animal house of our institute three weeks before the commencement of the experiment. The fishes were maintained in the large tank, and were fed twice daily with pelleted feed (Biomar, Nersac, France). 

### Isolation of c-type and g-type lysozyme cDNA and genomic DNA sequences

The full length cDNA for Asian seabass c-type and g-type lysozymes were obtained from cDNA libraries constructed in a previous study [26,31]. The cDNA sequences of the two genes were submitted to NCBI (Accession no, c-type: KF183643 and g-type: ABV66069). Analysis of ORF of the cDNA sequences was performed by using a web service ORF Finder (http://www.ncbi.nlm.nih.gov/projects/gorf/). The putative signal peptides were analysed using SignalP 3.0 Server (http://www.cbs.dtu.dk/services/SignalP). The molecular weight (MW) and theoretical isoelectric point (pI) of the deduced amino acid sequences were analysed using the export protein analysis program (http://au.expasy.org/tools). 

To obtain the genomic sequence of the two genes, primers were designed to amplify genomic DNA. The forward primers were located in the 5′ UTR and the reverse primers were in the 3′ UTR (see primer sequences in Table 1). PCR was performed in a volume of 25 μl consisting of 10 ng of genomic DNA, 1×PCR buffer with 1.5 mM MgCl_2_, 0.2 μM of each primer, 50 μM of each dNTP and 1 unit of Taq DNA Polymerase (Fermentas, PA, USA). The PCR program consisted of the following steps: 94°C for 2 min followed by 38 cycles of 94°C for 30 s, 55°C for 30 s and 72°C for 40 s, then a final step of 72°C for 5 min. The PCR products were cleaned using NucleoSpin Extract II Column (Macherey-Nagel, Düren, Germany) and cloned into the pGEM-T vector, and then sequenced using M13 and M13R primers and Bigdye chemicals on an ABI 3730xl DNA sequencer (Applied Biosystems, CA, USA). The full-length genomic DNA sequences of the c-type and g-type lysozyme genes were obtained by assembling sequences using software Sequencher 4.9 (Gene Codes, MI, USA) and submitted to NCBI (Accession no, c-type: KC460318; g type: KF183642).

**Table 1:** Primers used for amplifying the cDNA and genomic DNA of c-type and g-type lysozymes and expression analysis in Asian seabass.

Primer name	Primer sequence (5′-3′)	Annealing temperature (°C)	Application
(c-type)-A	GCAGCGAGCTTCTGACTGATGAT	60	cDNA 3′ RACE
(c-type)-B	TTACACCCCACAACCTGACACATA	60	cDNA 5′ RACE
g(c-type)-F	ACATTTGATCCAGCAGAGAATACA	57	Amplified genomic DNA
g(c-type)-R	CACCCCACAACCTGACACA		
r(c-type)-R	GCAGCGAGCTTCTGACTGATGAT	55	Real-time PCR
r(c-type)-F	ATTACACCCCACAACCTGACACATAG		
e(c-type)-F	CCGAATTCCATGAGGGGTCTGCT	57	Expression in *E. coil*
e(c-type)-R	CGAGCTCAGTTACACCCCACAACC		
snp(c-type)-F	CATACTTATTAGCGTCCTTGC	55	SNP PCR
snp(c-type)-R	CTATCTACAGCCACATCCACC		
(g-type)-A	AGAGTCCAGGGCTGGAAAT	60	cDNA 3′ RACE
(g-type)-B	TGTACCACTGAGCTCTGGC	60	cDNA 5′ RACE
g(g-type)-F	ATGGGTGGGTGCTTTTACCTTCTT	57	Amplified genomic DNA
g(g-type)-R	AGCCGTTGCTTTTGTACCACTGAG		
r(g-type)-F	AGAGTCCAGGGCTGGAAAT	55	Real-time PCR
r(g-type)-R	TGTACCACTGAGCTCTGGC		
e(g-type)-F	CGAATTCCATGGGTTATGGAGACATCA	58	Expression in *E. coil*
e(g-type)-R	TCTCGAGCTAAAAGCCGTTGCTTTTG		
snp(g-type)-F	TTGCGATAATGACTGACTAATG	55	SNP PCR
snp(g-type)-R	TCAACCTACAGAAATGGGAAT		

Note: Restriction site is underline

### Phylogenetic analysis

To evaluate the evolutionary relationships between the two lysozyme genes in Asian seabass and those in other species, we retrieved cDNA sequences of lysozyme genes of the other species (see details in Figure S1) from Genbank. The protein sequences were aligned using the Clustal X 1.83 program [34]. Phylogenetic analysis was carried out using the MEGA package [35] with neighbour-joining bootstrap tests (10,000 times). Genetic distances among protein sequences were evaluated with Poisson correction using MEGA. 

### Analysis of the expressions of the c-type and g-type lysozyme genes using qRT-PCR

Total RNA was isolated from five fishes at the age of three months using Trizol reagent (Invitrogen, CA, USA) according to the manufacturer’s instructions in the following tissues: heart, liver, spleen, gill, kidney, muscle, intestine and brain. RNA was transcribed with MMLV reverse transcriptase (Clontech, CA, USA) following the supplier’s protocol. The mRNA expressions of the c-type and g-type lysozyme genes of the Asian seabass in the different tissues were detected by real time quantitative PCR (qPCR) with the elongation factor 1-alpha (EF1α) gene as an internal control [13,31,36]. The c-type and g-type lysozyme cDNA from various tissues were amplified with the primers listed in Table 1. PCR amplification was conducted with an IQ5 (Bio-RAD, CA, USA) in a total volume of 15 μl containing 1×Maxima^TM^ SYBR Green qPCR Master Mix (Fermentas, PA, USA), 0.3 μl of each primer (10 pm/μl) and 1 μl (i.e., 100 ng) of template cDNA. The cycling conditions consisted of an initial, single cycle of 10 min at 95 °C followed by 40 cycles of 15 s at 95 °C, 30 s at 55 °C and 20 s at 72 °C. After completion of the qPCR, a melting-curve analysis was performed to confirm the specificity of the amplification. qPCRs were performed in triplicates. The expression levels of c-type and g-type lysozymes were analysed using ΔΔCT method [37]. 

### Examining temporal expressions of the c-type and g-type lysozymes in tissues after bacterial challenge

qRT-PCR were conducted to investigate the temporal and spatial expression patterns of the c-type and g-type lysozyme genes in Asian seabass challenged with *V. harveyi*, which is a significant pathogen of marine vertebrates and invertebrates. The 30 individuals maintained for three weeks in our fish facility were divided into two groups. Fifteen fishes were transferred into the test tank where each fish was injected intraperitoneally with 0.1 ml of *V. harveyi* (~*e* ^9^ copy/ml) in phosphate-buffered saline as described by Xia et al [31]. In the control tank, another 15 fishes received an intraperitoneal injection of 0.1 ml of phosphate-buffered saline. For every time point, three fishes [13,38] from each tank were sacrificed at 1, 3, 6, 12 and 24 h post-injection (hpi). The samples of spleen, kidney and liver were collected from each fish, preserved in Trizol reagent (Invitrogen, CA, USA), and stored at -80°C until use. The RNA extraction, cDNA synthesis, qRT-PCR thermal profile and the data analysis were conducted as described above. The normalized values for the test fishes challenged with the bacteria *V. harveyi* were compared with the control level at the respective time and tissues. The resulting expression ratio was then converted to natural logs and analysed with Cluster 3.0 (http://bonsai.ims.u-tokyo.ac.jp/~mdehoon/software/cluster/software.htm#ctv).

### Expression of the two lysozyme genes of Asian seabass in *E.coli* and purification of the recombinant proteins

A forward primer for the c-type and g-type lysozyme genes with an EcoRІ restriction site and a reverse primer with an XhoІ restriction site were designed, respectively, to amplify the coding sequence for the c-type and g-type lysozymes (Table 1). PCR amplifications were performed for one cycle of 3 min at 94°C, 30 cycles of 30 s at 94°C, 30 s at 57°C and 30 s at 72°C, with a final extension step of 5 min at 72°C. The PCR amplified c-type and g-type lysozyme gene fragments were digested with EcoRІ and XhoІ, ligated into the pGEX-4t-3 expression vector linearized with the same enzymes, and transformed into XL1-blue competent cells (Invitrogen, CA, USA). After sequencing the expression vector to ensure the correct inframe insertion, the DNA construct was transformed into the host BL21 (DE3) pLysS strain for protein expression. The fusion protein was expressed by isopropyl-beta-D-thiogalactopyta-noside (IPTG) induction, with the final concentration of 0.8 mM for induction, when the OD_600_ of the culture reached 0.6 in liquid LB medium. The cells were cultured at 25°C for 2 h post-induction and the cell suspension was subsequently gently sonicated on ice, and the recombinant lysozyme was released into the supernatant. The supernatant was collected and purified by Glutathione Sepharose 4B affinity chromatography (GE Healthcare, LC, UK). Half of the fusion proteins were digested with thrombin on the column before elution with PBS, whereas the remaining proteins were eluted from the column with glutathione. Purification and elution were performed according to the instruction manual (GE Healthcare, LC, UK). The protein was loaded into 12% SDS-PAGE and visualized after staining with Coomassie brilliant blue R-250. The concentration of the purified protein was determined using the Bradford Protein Assay Kit (Beyotime, JS, China). 

### Examining the antimicrobial activities of the recombinant lysozymes

The mature peptides of the c-type and g-type lysozymes were purified with affinity column and released from the fusion protein by digestion with thrombin. In this study, antibacterial testing was carried out using *V. harveyi*, *P. damselae* and *E.coli* DH5α, as *V. harveyi* and *P. damselae* are the major pathogens for Asian seabass. The assay was performed according to a modified method by Sun et al [39]. The bacteria were cultured at 30°C in the LB medium in a shaker to OD_600_ of 1.0, collected and diluted using nutrient broth to a density of 2×10^5^ CFU/ml. 12 columns were filled with 0.5 ml nutrient broth contain 2×10^5^ CFU/ml tested strains. 0.5 ml of recombinant proteins was diluted with 0.5 ml nutrient broth in column 1 and blended together. Then serial doubling dilutions in columns 2-10 were conducted. Column 11 served as a growth control, and column 12 was used as blank control. The tubes with *V. harveyi* and *P. damselae* were incubated at 30°C for 16 h, and the plates with *E.coli* were incubated at 37°C for 16 h. Bacterial density was measured according to an absorbance of 630 nm. The 50 mM phosphate buffer saline (pH 7.4) in which the recombinant proteins were dissolved was used as the control.

### Identification and genotyping of SNPs in the lysozyme genes

To identify SNPs in the lysozymes, two pairs of primers (Table 1) were designed to amplify the genomic DNA of 10 F_2_ Asian seabass individuals collected from an F_2_ Asian seabass population. PCR was conducted as described above. The genotyping of four SNPs were conducted by directly sequencing PCR products using an ABI 3730xl sequencer as described above. SNP genotypes were analysed by using the software Sequencher (Genecodes, MA, USA) 

### Analysis of the associations of the SNPs in the lysozyme genes with resistance to the big belly disease

In order to examine whether SNPs in the two lysozymes were associated with resistance to the big belly disease, we genotyped individuals before (as a control group) and after (as a disease-resistant group) an outbreak of the big belly disease in an Asian seabass population generated by a mass cross between 15 males and 15 females. After the outbreak of the disease at the age of the 25 days post hatch (dph), only less than 20% of fish survived at 90 dph. We regarded the surviving individuals after the outbreak of the disease as individuals resistant to the disease. We collected fin clips from 139 and 151 individuals before the outbreak of the disease for genotyping c-type and g-type, respectively, and 175 and 185 individuals at 90 dph (after the outbreak of the disease) for c-type and g-type, respectively. SNPs were genotyped in 314 and 336 fish for c-type and g-type, respectively. The Statistical Program for Social Science (SPSS) (SPSS Inc., IL, USA) version 12.0 was used for data analysis. The genotype frequency and allele frequency were compared in individuals collected before and after the outbreak of the big belly disease. Chi-square tests were conduct to analyse the significance level of the differences. 

## Results

### cDNA sequences of the two lysozyme genes in Asian seabass and evolutionary relationships among lysozyme genes

**Figure 1:**
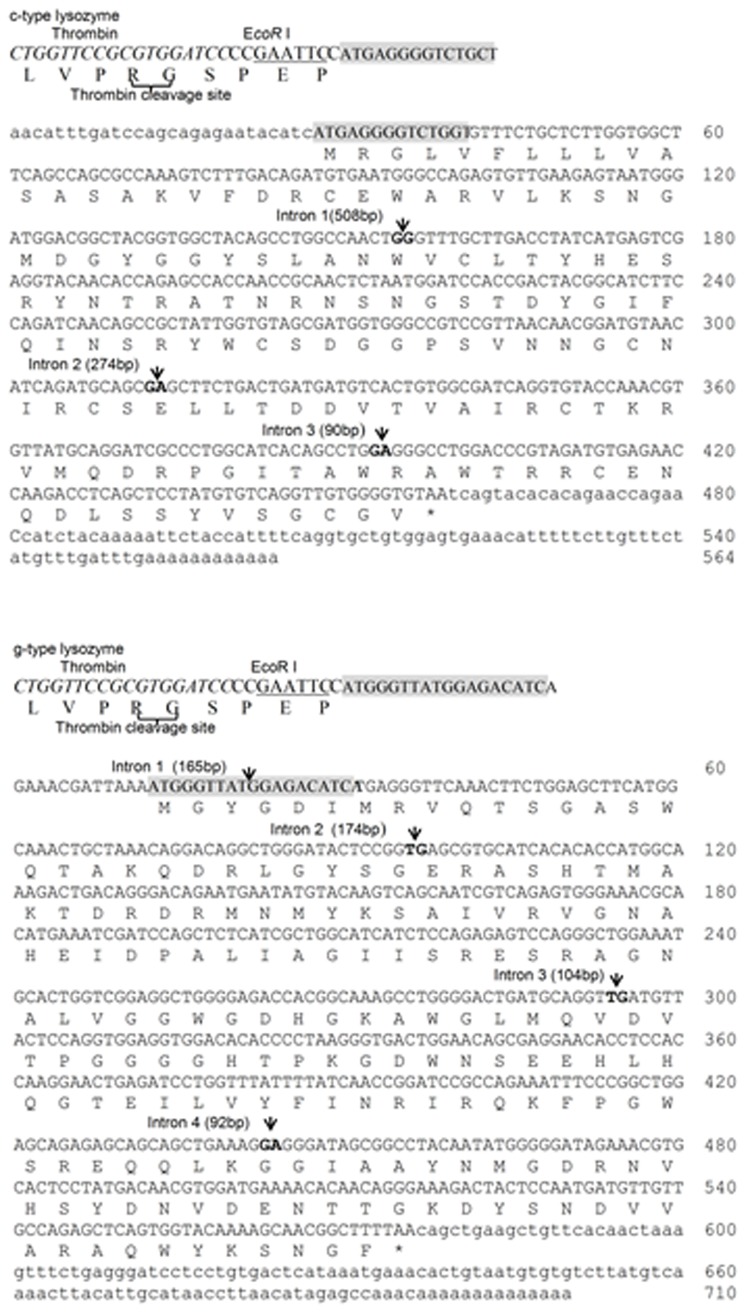
The full-length cDNA and the deduced amino acid sequences of the c-type and g-type lysozyme genes in Asian seabass. Intron positions are indicated by solid triangles; restriction endonucleases are indicated underline; the primers are greyed.

The full-length cDNA sequence of c-type lysozyme was 566 bp and contained an open reading frame (ORF) of 435 bp. The 5′-untranslated region (UTR) and 3′-UTR of the c-type lysozyme cDNA sequence were 27 bp and 104 bp, respectively. The c-type lysozyme was predicted to encode a polypeptide of 144 amino acid (aa) residues with the signal peptide comprising the first 15 residues (Figure 1). The mature peptide of c-type lysozyme had a calculated molecular mass of 16.02 kDa and isoelectric point (pI) of 8.85. Multiple sequence alignment of c-type lysozyme with other fish counterparts (Figure S1) revealed that the amino acids critical for the fundamental structure and function of the c-type lysozyme were highly conserved in the fish lysozyme, such as the lysozyme catalytic sites (Glu^50^, Asp^67^), the Ca^2+^ binding site (Thr^99^, Val^104^ and Gla^105^), and the motif “GSTDYGIFQINS” at positions 64-75 flanking the active Asp^67^ are also highly conserved among species. The full-length cDNA sequence of the g-type lysozyme of Asian seabass was 710 bp in length and contained an ORF of 564 bp encoding 187 amino acid residues. The 5′- UTR and 3′-UTR of the g-type lysozyme were 12 bp and 134 bp, respectively. Typical signal peptide was not found. The calculated molecular mass of the g-type lysozyme was 20.73 kDa, with a pI of 7.99. In silico analyses revealed that the g-type lysozymes possesses a Goose Egg White Lysozyme (GEWL) domain (residues 16 to 185) containing three conserved catalytic residues Glu^71^, Asp^84^ and Asp^95^. Multiple sequence alignment of the g-type lysozyme with other fish counterparts (Figure S1) revealed that the amino acids critical for the fundamental structure and function of the g-type lysozyme were highly conserved in the fish lysozymes, such as the lysozyme catalytic sites and N-acetyl-D-glucosamine binding site (Gly^71^, Asp^95^, Pro^98^, His^102^, Ile^121^, Tyr^149^, Asn^150^ ).

A phylogenetic tree (Figure S2) was constructed for the sequences of the lysozymes from Asian seabass, other fish species and mammals using the neighbor-joining method. The c-type lysozyme of Asian seabass was closely related to the one in Senegalese sole, *Solea senegalensis* and rainbow trout, *Oncorhynchus mykiss*. The g-type lysozyme of Asian seabass was located in the same branch as the Mandarin fish, *Siniperca chuatsi*, and also closely related to the g-type lysozyme of European seabass, *Dicentrarchus labrax* and the Senegalese sole.

### Genomic structure of the two lysozyme genes in Asian seabass

The genomic sequence of the c-type lysozyme gene from the transcriptional start site to the transcriptional end site consisted of 1491 bp, comprising of four exons (157, 158, 80, 171 bp, respectively) and three introns (Accession no KC460318). The genomic DNA sequence of the g-type lysozyme gene consisted of 1232 bp, comprising of five exons (22, 75, 198, 149, 253 bp, respectively) and four introns (Accession no. KF183642).

### Expression profiles of the c-type and g-type lysozyme genes

**Figure 2:**
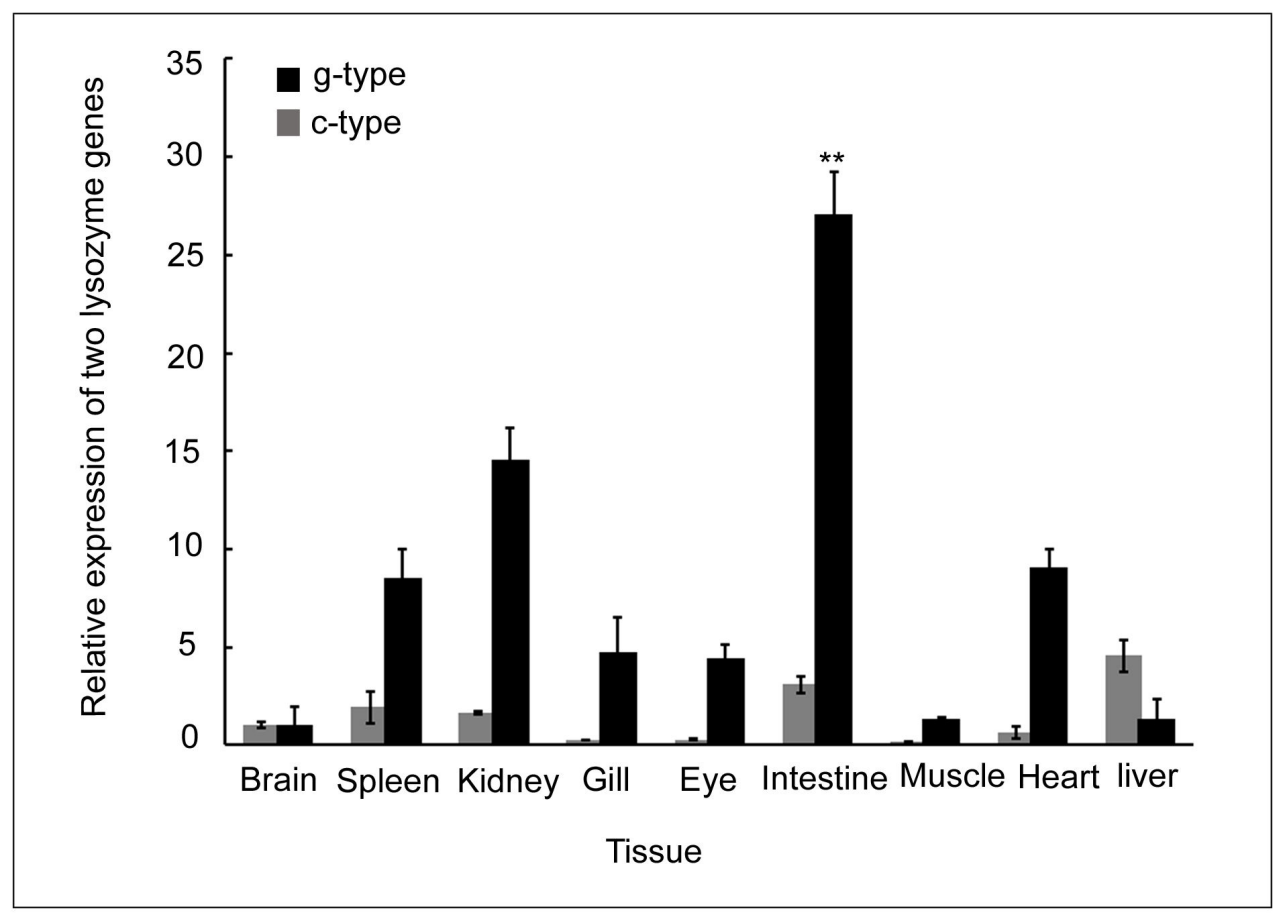
Tissue distribution of the c-type and g-type lysozyme genes in Asian seabass. The transcripts of genes were measured by qRT-PCR. The tissues, including liver, brain, spleen, kidney, gill, eye, intestine, muscle and heart were collected from five individual *Lates* *carcalifer*. **: *p* < 0.01.

Expressions of the c-type and g-type lysozymes were detected in all tested tissues of untreated individuals (Figure 2). The highest expression levels were detected in liver for the c-type lysozyme and intestine for the g-type lysozyme. Expression levels of the c-type lysozyme in liver and spleen were 4.5-fold and 1.65-fold higher than in the brain, respectively. Lower expression levels (<1.0-fold of brain) were determined in other tissues. The g-type lysozyme mRNA levels in intestine and kidney were 27.0-fold and 14.5-fold higher than in brain, respectively. A high mRNA level was also detected in heart and spleen (9-fold and 8.5-fold higher than in brain, respectively) (*p* < 0.05).

### Temporal expression profiles of c-type and g-type lysozyme genes post *V. harveyi* injection

**Figure 3:**
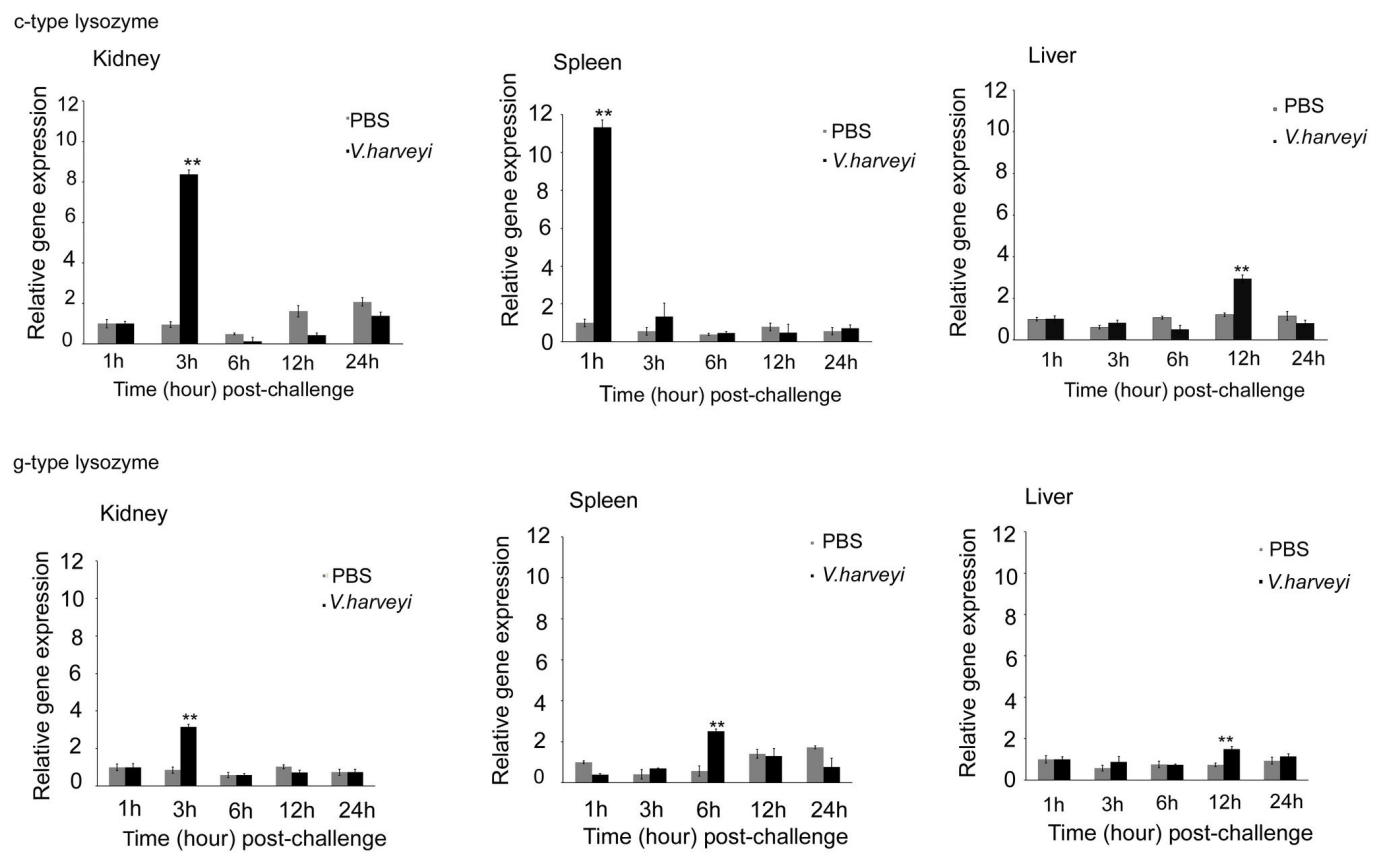
Relative expression levels of the c-type and g-type lysozymes of Asian seabass challenged with *V*. ***harveyi***. The expressions of c-type and g-type lysozymes were determined in kidney, spleen and liver by qRT-PCR method. The samples were analyzed at 1h, 3h, 6h, 12h and 24h post-treatment. Expression of EF1α was as an internal control for qRT-PCR. Each experiment was performed at least in triplicate. Data are shown as mean ± SE (n = 3). **:*p* < 0.01.

In order to investigate a potential immunological function and the response to bacteria for both lysozyme types, the temporal expressions of the c-type and g-type lysozymes in liver, spleen and kidney of the Asian seabass challenged with *V. harveyi* were measured by qRT-PCR (Figure 3). Controls were injected with PBS. EF1α was employed for normalization and PBS control at 1 h as calibrator. After the challenge with *V. harveyi*, the c-type lysozyme expression level in the kidney increased up to 11.3-fold at 1 h, 8.4-fold in spleen at 3 h and 2.9-fold in liver at 12 h (*p* < 0.05), which was the highest expression level among the described tissues. As time progressed, the expression of the c-type lysozyme in the challenge group dropped back to the normal level, without significant difference compared with the control. The gene expression showed similar profiles in liver, kidney and spleen. The expression levels of g-type lysozyme were up-regulated drastically and then decreased to the normal level. The peak value appeared at 3 h with 3.2-fold increase in kidney, 6 h with 2.5-fold in spleen, and 12 h with 1.5-fold in liver (*p* < 0.05). These data indicate that c-type and g-type lysozyme gene may contribute to the innate immune defence upon bacterial infection.

### Recombinant expression, purification and antimicrobial activity assay of c-type and g-type lysozymes

**Figure 4:**
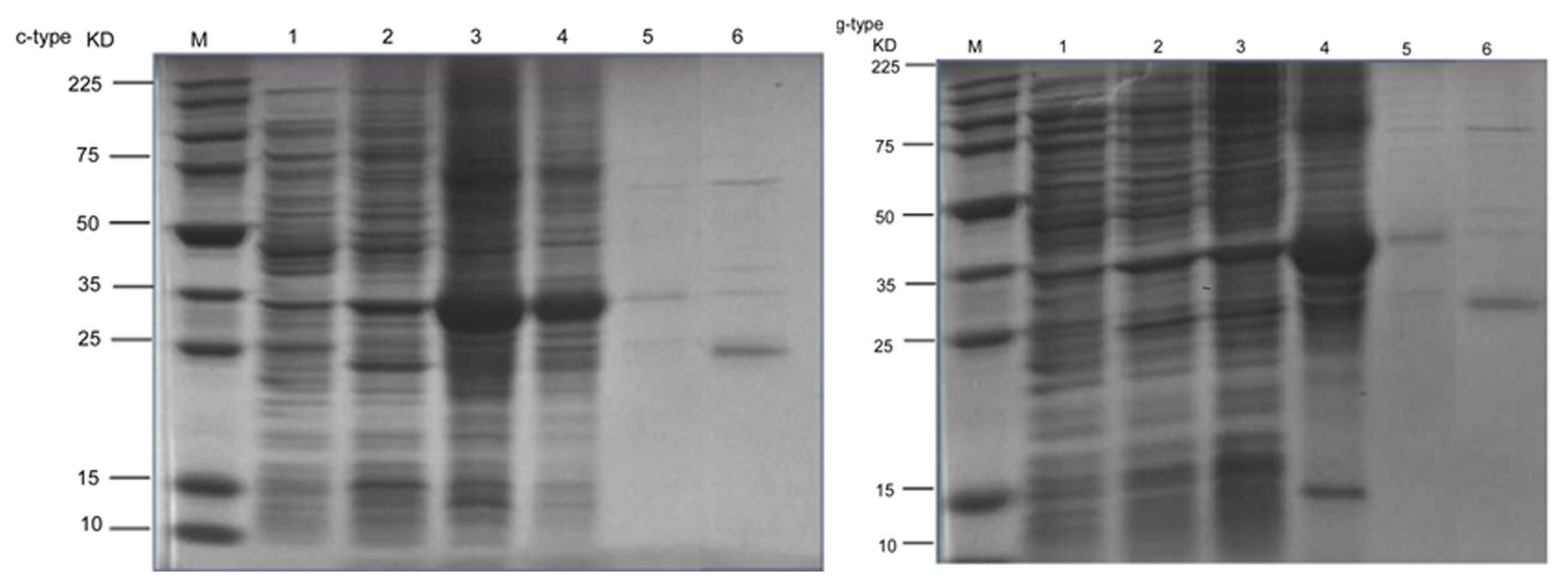
Expression and purification of Asian seabass c-type and g-type lysozymes. Lane M: protein molecular standard; lane 1: pGEX-c/g-type lysozyme in BL21(DE3) without IPTG induction (supernatant); lane 2: pGEX-c/g-type lysozyme in BL21(DE3) without IPTG induction (sediment); lane 3: pGEX-c/g-type lysozyme in BL21(DE3) with IPTG induction (supernatant); lane 4: pGEX-c/g-type lysozyme in BL21(DE3) with IPTG induction (sediment); lane 5: purified fusion GST-c/g-type lysozyme; lane 6: GST and mature peptide of c/g-type lysozyme released by digestion with thrombin.

The recombinant plasmid pGEX-4t-3 was transformed and expressed in *E. coli* BL21 (DE3) as described above. After IPTG induction, the whole cell lysate was analysed by SDS-PAGE. The lysozyme protein gave a distinct band at about 36 kDa (GST-protein 20 kDa + c-type mature peptides 16 kDa) for c-type lysozyme and 40.13 kDa (GST-protein 20 kDa + g-type mature peptides 20.13 kDa) for g-type lysozyme as compared with the control (Figure 4). It was in accordance with the predicted molecular mass computed from the amino acid sequence. 

**Figure 5:**
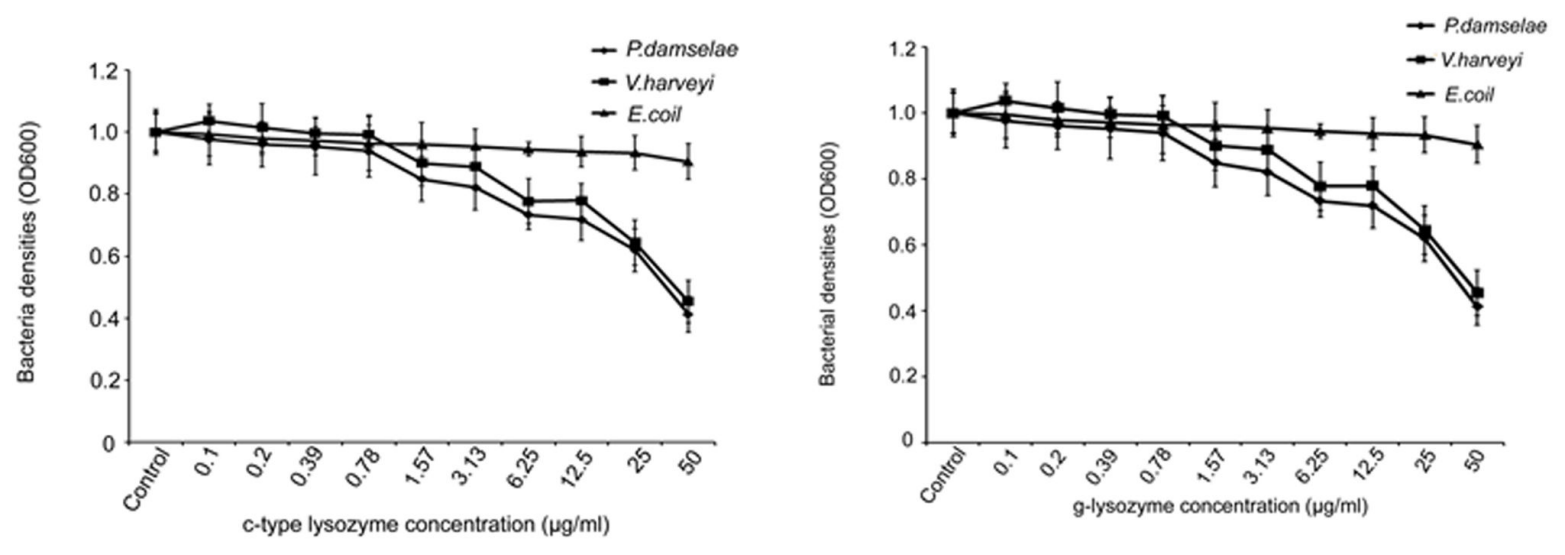
Anti-bacterial activities of the recombinant c-type and g-type lysozymes of Asian seabass. The results represent the mean values with standard deviations (n=6).

In order to test the antimicrobial activities of the c-type and g-type lysozymes, a bacteria growth inhibition assay was employed and three types of bacteria were used. Our results showed that high concentration (> 6.25 µg/ml mature peptide) of the c-type and g-type lysozymes could inhibit the growth of *V. harveyi* and *P. damselae*, and indicate that two recombinant lysozymes work against bacterial pathogens in a dose-dependent manner (Figure 5). Meanwhile, two types of lysozymes did not effectively inhibit the growth of *E.coli*. The results indicate that the c-type and g-type lysozymes can effectively inhibit bacterial pathogens. 

### Associations between SNPs in the two lysozyme genes and resistance to big belly disease in Asian seabass

Two SNPs were identified in each of the two lysozyme genes. In the c-type lysozyme, one (SNPc1, genomic DNA position 655, C/T) was located in exon 2, and the other (SNPc2, genomic DNA position 934, C/T) was in intron 3. In the g-type lysozyme, two SNPs were located in exon 1 (SNPg1, genomic DNA position 235, A/C; SNPg2, genomic DNA position 237, A/G). SNPc1 in c-type lysozyme and SNPg1, SNPg2 in g-type lysozyme were found in exon sequences, and resulted in amino acid changes. The allele and genotype frequencies of the four c-type and g-type SNPs are shown in Table 2. For SNPc1, genotypes CC and CT were detected with frequencies of 89.9% and 10.1% in the control group, respectively. TT was only detected in two samples out of 175 individuals. In the disease-resistant group, genotypes CC, CT and TT were detected with frequencies of 82.3%, 16.6% and 1.1%, respectively. For SNPc2, the frequencies of CC, CT and TT in the control group were 15.5%, 39.1% and 45.4%, respectively, while the frequencies of CC, CT and TT were 12.2%, 28.8% and 60% in the disease resistant group, respectively. There were significant differences in genotype frequency between the control and resistant groups of the c-type lysozyme. For SNPg1, genotype AA, AC and CC were detected with frequencies of 70.9%, 17.2% and 11.9% in the susceptible group, while the genotype frequencies were 63%, 23.4% and 13.6% in the resistant group, respectively. For SNPg2, the frequencies of AA, AG and GG were 74.2%, 23.2% and 2.6%, respectively in the susceptible group, while the genotype frequencies were 68.8%, 30.3% and 1.1% respectively in the resistant group. Allele frequencies at the four SNP loci showed significant differences (*p* < 0.05) between individuals before and after the outbreak of the big belly disease. The four SNP alleles C, T, A and G at the four SNP loci (Table 2) were frequently observed in disease resistant individuals in contrast to the control individuals, suggesting that they might be related to the resistance to the big belly disease.

**Table 2:** Distribution of genotypes and allele frequency for each SNP locus in two lysozyme genes in control and disease resistance groups of Asian seabass.

**SNP**	**Genotype (individual No.)**			**Allele frequency (Number)**			
	**Control**	**Disease resistance**	***X^2^***	**Allele**	**Control**	**Disease resistance**	***X^2^***
c-type SNP1							
CC (n=269)	125	144	8.48	C	364	257	24.83
CT (n=143)	114	29	(*p*<0.05)	T	114	33	(*p*<0.005)
TT (n=2)	0	2					
c-type SNP2							
CC (n=44)	27	17	13.04	C	122	74	10.08
CT (n=108)	68	40	(*p*<0.005)	T	226	204	(*p*<0.05)
TT (n=161)	79	82					
g-type SNP1							
AA (n=223)	107	116	6.06	A	232	257	8.69
AC (n=69)	18	25	(*p*<0.05)	C	70	111	(*p*<0.05)
CC (n=43)	26	43					
g-type SNP2							
AA (n=239)	112	127	6.49	A	259	310	6.04
AG (n=91)	35	56	(*p*<0.05)	G	43	60	(*p*<0.05)
GG (n=6)	4	2					

*p* < 0.05 was considered to be statistically significant.

## Discussion

We identified two lysozyme genes from the Asian seabass. A phylogenetic analysis of the amino acid sequences revealed that the two genes were the c-type and g-type lysozyme genes from Asian seabass. We further analyzed their molecular characteristics, expression and regulation after bacterial infections. The predominant molecular characteristics of c-type and g-type are as follows. Firstly, the c-type lysozyme in Asian seabass, containing the deduced 143 amino acid residues, is similar to other fish species, such as, rainbow trout [16], Nile tilapia [40] and grass carp [13]. The g-type lysozyme in Asian seabass, containing 186 deduced amino acid residues, is similar to those in common carp [17] and grass carp with 185 residues[13]. In contrast, some fish species possess longer g-type lysozyme proteins of 193-195 amino acid residues, such as Japanese flounder [14], orange-spotted group [11], larger yellow croaker [12], mandarin fish [39], brill [3] and turbot [15], while the Atlantic cod has a g-type lysozyme with a longer deduced lysozyme protein containing 217 aa [41]. Although different fish species contain the g-type lysozyme cDNA of different ORF lengths, the conserved domain of that deduced g-type lysozyme protein was much similar. The genomic structure analysis revealed that the c-type lysozyme gene consisted of four exons and three introns that are consistent with other fish species [10,42]. The genomic structure analysis revealed that the g-type lysozyme gene of Asian seabass was organized into five exons and four introns. The structure and exon-intron boundaries were well conserved with respect to other fish [12,39,41]. 

In fish and mammals, the presence of two putative conserved catalytic residues (Glu and Asp) in c-type lysozyme are critical for the lysozyme lytic activity to bacterial cell wall [43]. The c-type lysozyme of Asian seabass also possessed these putative conserved cysteine residues (Glu and Asp). The g-type lysozyme in most of the known fish species contains three conserved catalytic residues (Glu^71^, Asp^84^ and Asp^95^), except the common carp in which the second catalytic residue Asp^86^ was replaced by proline [17]. G-type lysozyme in Asian seabass possesses a GEWL domain with conserved residues essential for catalytic activity, which exists in most fish species, bivalves as well as birds and mammals [8,44]. However, it has been discovered that g-type lysozyme in molluscs shared one conserved cysteine with those from birds and mammals, and six conserved cysteines were observed in molluscs g-type lysozymes, with two unique cysteines in the g-type lysozyme of *O. hupensis* [18]. Thus, the conserved residues in the lysozymes of Asian seabass are expected to have similar catalytic activity with those of other fish and avian species. 

We examined the expressions of the c-type and g-type lysozymes in different tissues. The c-type lysozyme was highly expressed in liver, intestine, spleen and kidney, but expression was low in other analyzed tissues. Similar expression of the c-type lysozyme was seen in zebrafish [45] and rainbow trout [16]. Since liver, spleen and kidney are important organs for immune functions in fish, these data suggests that c-type lysozyme genes might play an important role in disease resistance. The g-type lysozyme was expressed in all analyzed tissues and highly expressed in intestine, kidney and spleen. A similar expression pattern of the g-type lysozyme was observed in Japanese flounder [14], mandarin fish [39], grass carp [13] and yellow croaker [12]. However, the expression of this gene in mammals and birds were restricted in some tissues compared to that in fish. For instance, the g-type lysozyme was found only in the cells of bone marrow and lungs in chickens [46]. Similarly, low levels of g-type lysozyme were expressed in the adult kidneys of mammals [47]. This expression difference between fish and land animal species may be related to an alteration of biological function during their evolution. Besides that, we observed that the g-type lysozyme in Asian seabass was highly expressed in the intestine, suggesting that, besides its role in defense against bacterial pathogens, this gene may also play a significant role in digestion [4].

In this study, the temporal expression patterns of the c-type and g-type lysozymes in liver, kidney and spleen after *V. harveyi* challenge was recorded to further investigate their functions. The results indicate that the c-type and g-type lysozyme genes were up-regulated in some tissues when challenged with the pathogen, *V. harveyi*. The strongest up-regulation of c-type lysozyme mRNA levels was detected in both kidney and spleen (Figure 3), but low expression level was detected in the liver. Among these tissues, spleen and kidney are systemic immune organs and liver is the detoxification organ in fish. These results suggest that c-type lysozymes play a direct role in defense against invading pathogens. The up-regulation of mRNA varied between the c-type and g-type lysozymes under the same treatment conditions and tissue types, suggesting different functions between these two genes. Similar up-regulation in the expression of these two genes after the bacterial challenge were reported in a number of fish species, such as orange-spotted grouper [11] , Japanese flounder [14] and half-smooth tongue sole [48]. The expression of the c-type lysozyme was up-regulated by different bacterial stimuli in the kidney of the orange-spotted grouper [49], however no significant change was observed in brill after injection of LPS or bacteria [2]. The g-type lysozyme transcription level in blood, liver, spleen and head kidney was up-regulated after challenge with the bacteria *Vibrio anguillarum* in half-smooth tongue sole [48]. In grass carp, the mRNA level of both genes were up-regulated after being challenged with *Aeromonas hydrophila* [13]. In flatfish brill inoculated with LPS and *P. damselae* *subsp.* *piscicida*, the g-type transcripts increased but no induction was observed for the c-type lysozyme in the head kidney, suggesting an important defensive role for the g-type lysozyme in fish [3]. However, in our research, the c-type lysozyme mRNA level in the kidney and spleen was much higher than that of g-type lysozyme, suggesting an important role of the c-type lysozyme in defense against invading pathogens [13]. The expression level of both lysozyme genes increased after being challenged with pathogens, suggesting that they participate in the immune response. 

In this study, the recombinant c-type and g-type lysozymes of Asian seabass had antimicrobial activities against fish pathogenic bacteria. Recombinant lysozymes also had lytic activities in other fish species. For example, Japanese flounder recombinant c-type lysozyme in the baculovirus expression system showed lytic activities [50]. Studies using the orange-spotted grouper recombinant c-type lysozyme showed that this protein has antibacterial activities against *M. lysodeikticus*, *V. alginolyticus*, *S.* aureus and *S. iniae* [49]. Recombinant g-type lysozyme of mandarin fish in the *E. coli* expression system showed lytic activity against the bacteria *M. lysodeikticus*, *V. alginolyticu* and *A. hydrophila* [8,51]. In rock bream, the recombinant g-type lysozyme showed lytic activity against *Vibrio salmonicida*, *Listeria monocutogenes* and *Micrococcus lysodeikticus* [52]. In grass carp, both recombinant g-type and c-type lysozyme possessed lytic activities against fish pathogens [13]. In invertebrate, the recombinant g-type lysozyme in mollusc inhibit the growth of both Gram-positive and Gram-negative bacteria [8]. These evidences strongly suggest that both lysozymes play an important role in the defense against bacterial infection. 

Four SNPs were detected in these two lysozyme genes of Asian seabass. Analysis of allele frequencies in the control and the big belly resistant groups revealed significant differences of allele frequencies, suggesting that the SNPs may be associated with the resistance to big belly disease in Asian seabass. In other species, polymorphisms in lysozyme genes were also found to be associated with resistance to bacterial diseases [24,53]. For example, the polymorphism of the lysozyme gene in Zhikong scallop (*Chlamys farreri*) was associated with susceptibility/resistance to *Listonella anguillarum* [53]. In the clam *Meretrix meretrix*, SNPs in the i-type lysozyme gene was associated with *Vibri*o resistance [24]. The four SNPs detected in this study may be useful in the selection of Asian seabass resistant to the big belly disease. Certainly, it is essential to examine the associations in more populations before they can be used in breeding for resistance against the big belly disease. We have noticed that three of the four SNPs detected in the c- and g-type lysozymes changed the sequences of amino acids. It is interesting to examine whether the associations between the polymorphisms and the resistance to big belly disease are caused by the polymorphisms themselves or by their linkage to other genes. It could also be interesting to examine whether the SNPs in the two lysozyme genes in Asian seabass are associated with resistance to other bacterial pathogens or even viral pathogens, and also with growth related traits [23].

In conclusion, the c-type and g-type lysozymes were identified and characterized in the Asian seabass. The expression levels in various tissues after being challenged with *V. harveyi* and *P. damselae* suggest that these two lysozymes are important in the defense against invading bacterial pathogens. The purified recombinant proteins of these two lysozyme genes show obvious antibacterial activities. We also showed significant associations (*p* < 0.05) between lysozyme polymorphisms and resistance against the big belly disease. This study provides new insights on the antibacterial functions of the c-type and g-type lysozymes. The four SNPs in the two genes associated with the resistance to bacterial pathogens may facilitate the selection of Asian seabass resistant to the big belly disease. 

## Supporting Information

Figure S1 **Alignment of amino acid sequence of lysozyme homologs.** Left side: “*” indicates the identical amino acid residues; (: or .) are indicates similar residues. Lysozyme catalytic residues Glu (E) and Asp (D) are bold; lysozyme catalytic cleft is gray with underline; Ca^2+^ binding site is boxed; N-acetyl-D-glucosamine binding site is gray; conserved motif is black shading. c-type lysozymes: Human (*Homo sapiens*), AAI00886; Mouse (*Mus musculus*), NP_059068; Pig (*Sus scrofa*), AAB16862; Rainbow trout (*Oncorhynchus mykiss*), AF321519_1; Medaka (*Oryzias latipes*), ACO82287; Nile tilapia (*Oreochromis niloticus*), XP_003457546; Fugu rubripes (*Takifugu rubripes*), NP_001027914.1; Yellow perch (*Perca flavescens*), ACO34809; Senegalese sole (*Solea senegalensis*), ABC49680; Asia seabass (*Lates calcarifer*), KF183643.
Right side: “*” indicates the identical amino acid residues; (: or .) indicates similar residues. Lysozyme catalytic residues Glu (E) and Asp (D) are bold; lysozyme catalytic cleft is gray with underline; Ca^2+^ binding site is boxed; N-acetyl-D-glucosamine binding site is gray; conserved motif is black shading. **g-type** lysozymes: Human (*Homo sapiens*), AAI00886; Mouse (*Mus musculus*), AAI47568.1; Asia seabass (*Latescal carifer*), ABV66069; European seabass (*Dicentrarchus labrax*), CBJ56263; Rainbow trout (*Oncorhynchus mykiss*), ACO08589; Blue catfish (*Ictalurus furcatus*), ADO28271; Gilt-head bream (*Sparus aurata*), CAO78618; Fugu rubripes (*Takifugu rubripes*), NP_001027764; Senegalese sole (*Solea senegalensis*), BAG14278; Mandarin fish (*Siniperca chuatsi*), AAU86896.
(TIF)

Figure S2 **A NJ-phylogenetic tree of c-type and g-type lysozyme genes.**
(TIF)
