# Supplementary figures and images for: Analysis of Two Lysozyme Genes and Antimicrobial Functions of Their Recombinant Proteins in Asian Seabass
Source: PLoS One. 2013 Nov 7;8(11):e79743. doi: 10.1371/journal.pone.0079743 (PMC3820614; doi:10.1371/journal.pone.0079743)

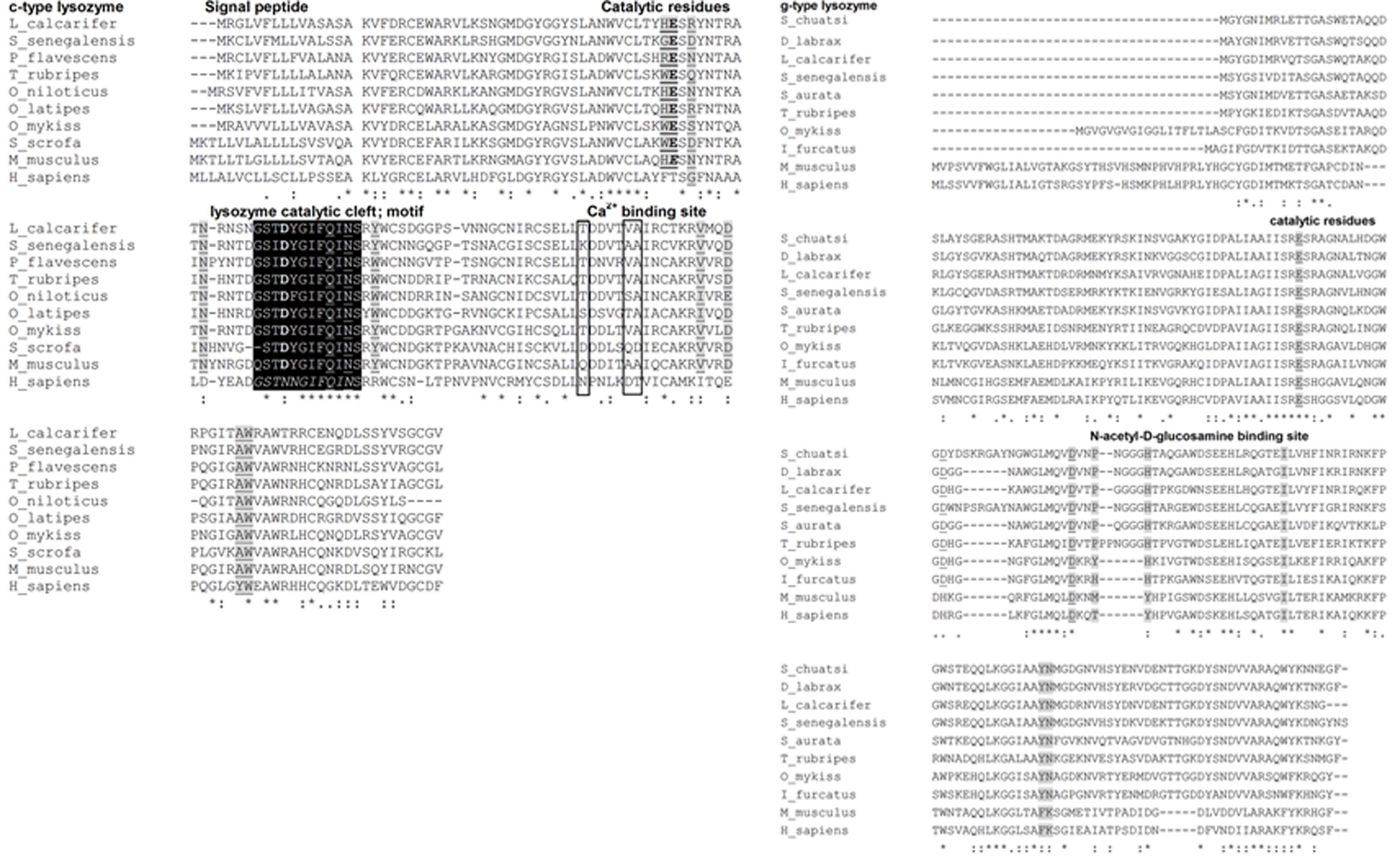

Supplement: Figure S1 — Alignment of amino acid sequence of lysozyme homologs. Left side: “*” indicates the identical amino acid residues; (: or .) are indicates similar residues. Lysozyme catalytic residues Glu (E) and Asp (D) are bold; lysozyme catalytic cleft is gray with underline; Ca2+ binding site is boxed; N-acetyl-D-glucosamine binding site is gray; conserved motif is black shading. c-type lysozymes: Human (Homo sapiens), AAI00886; Mouse (Mus musculus), NP_059068; Pig (Sus scrofa), AAB16862; Rainbow trout (Oncorhynchus mykiss), AF321519_1; Medaka (Oryzias latipes), ACO82287; Nile tilapia (Oreochromis niloticus), XP_003457546; Fugu rubripes (Takifugu rubripes), NP_001027914.1; Yellow perch (Perca flavescens), ACO34809; Senegalese sole (Solea senegalensis), ABC49680; Asia seabass (Lates calcarifer), KF183643. Right side: “*” indicates the identical amino acid residues; (: or .) indicates similar residues. Lysozyme catalytic residues Glu (E) and Asp (D) are bold; lysozyme catalytic cleft is gray with underline; Ca2+ binding site is boxed; N-acetyl-D-glucosamine binding site is gray; conserved motif is black shading. g-type lysozymes: Human (Homo sapiens), AAI00886; Mouse (Mus musculus), AAI47568.1; Asia seabass (Latescal carifer), ABV66069; European seabass (Dicentrarchus labrax), CBJ56263; Rainbow trout (Oncorhynchus mykiss), ACO08589; Blue catfish (Ictalurus furcatus), ADO28271; Gilt-head bream (Sparus aurata), CAO78618; Fugu rubripes (Takifugu rubripes), NP_001027764; Senegalese sole (Solea senegalensis), BAG14278; Mandarin fish (Siniperca chuatsi), AAU86896. (TIF) [file pone.0079743.s001.tif]

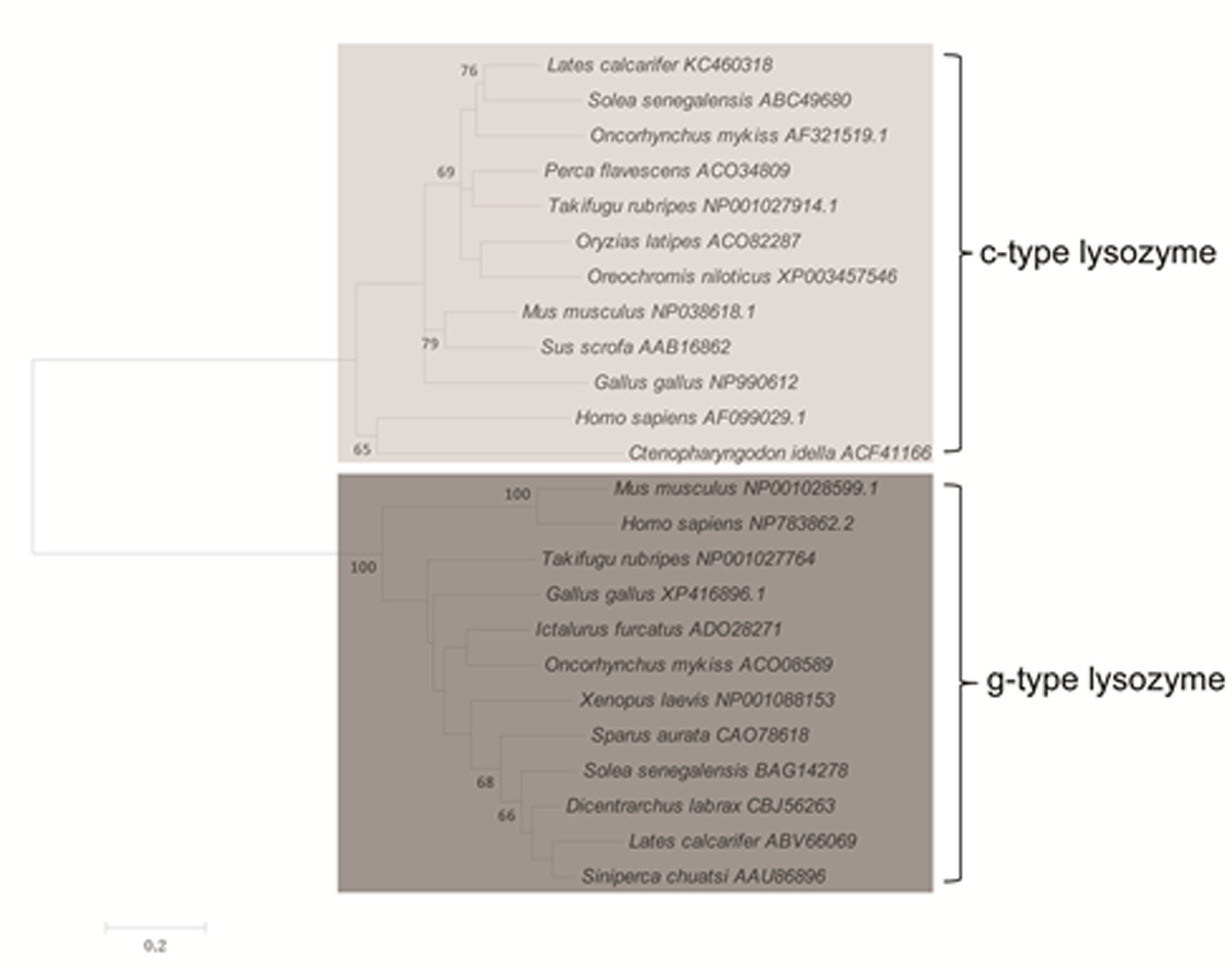

Supplement: Figure S2 — A NJ-phylogenetic tree of c-type and g-type lysozyme genes. (TIF) [file pone.0079743.s002.tif]
